# Oral bioavailable ITRI-148 degrades androgen receptor variants and overcomes antiandrogen resistance in advanced prostate cancer

**DOI:** 10.1016/j.neo.2025.101253

**Published:** 2025-11-13

**Authors:** Chiu-Lien Hung, Wen-Ning Hsu, Tsan-Chun Wang, Wan-Ru Chen, Yu-Ting Chen, Zong-Keng Kuo, Tsan-Lin Hu, Yu-Chin Lin, Hsun-Hao Yeh, Han-Chen Lin, Chia-Jung Yu, Chih-Wei Fu, Hao-Hsuan Liu, Hung-Chih Hsu, Po-Hung Lin, See-Tong Pang, Chih-Ho Lai, Ling-Yu Wang

**Affiliations:** a Department of Preclinical Drug Discovery Technology, Biomedical Technology and Devices Research Labs, Industrial Technology Research Institute, Hsinchu 31040, Taiwan; b Department of Biochemistry and Molecular Biology, Chang Gung University, Taoyuan 33302, Taiwan; c Graduate Institute of Biomedical Sciences, Chang Gung University, Taoyuan 33302, Taiwan; d Department of Cell and Molecular Biology, Chang Gung University, Taoyuan 33302, Taiwan; e Molecular Medicine Research Center, Chang Gung University, Taoyuan 33305, Taiwan; f Department of Thoracic Medicine, Chang Gung Memorial Hospital at Linkou, Taoyuan 33305, Taiwan; g Division of Hematology-Oncology, Chang Gung Memorial Hospital at Linkou, Taoyuan 33305, Taiwan; h School of Medicine, College of Medicine, Chang Gung University, Taoyuan 33305, Taiwan; i Division of Urology, Department of Surgery, Chang Gung Memorial Hospital at Linkou, Taoyuan 33305, Taiwan; j Department of Microbiology and Immunology, Chang Gung University, Taoyuan 33302, Taiwan

**Keywords:** AR, AR-V7, Drug resistance, Prostate cancer, PROTAC

## Abstract

Androgen receptor (AR) signaling remains a key driver of castration-resistant prostate cancer (CRPC), with AR splice variants like AR-V7 contributing to resistance against second-generation antiandrogens. Targeting the AR N-terminal domain (NTD) provides a strategy to bypass ligand-binding domain (LBD)-mediated resistance. We developed ITRI-148, a CRBN-based AR-NTD degrader incorporating a rigid piperidine-alkyne linker optimized for oral pharmacokinetics. ITRI-148 efficiently degrades full-length AR, AR-V7, and clinically relevant mutants (L702H, H875Y). It facilitates the recruitment of active AR species to CRBN in the nucleus, promoting their polyubiquitination and proteasomal degradation. In CRPC and enzalutamide-resistant models, ITRI-148 robustly suppresses AR signaling and inhibits cell viability, outperforming enzalutamide. With long-term treatment, it achieves sustained AR suppression without inducing compensatory AR-V7 upregulation or PSA re-expression. In vivo, ITRI-148 demonstrates potent antitumor efficacy in both castrated and hormone-intact CRPC models, supported by favorable pharmacokinetic properties, stability and safety profiles. These findings position ITRI-148 as a promising next-generation AR-targeting agent capable of degrading resistant AR variants and providing durable inhibition of AR signaling in advanced prostate cancer.

## Highlights

• AR-NTD-targeting ITRI-148 degrades AR, AR-V7, and AR mutants via CRBN in CRPC cells.
• ITRI-148 surpasses enzalutamide and ARV-110 in suppressing AR-V7 expressing CRPC cell viability.
• ITRI-148 outperforms enzalutamide, providing durable AR signaling suppression in vitro.
• Rigid-linker design enhances ITRI-148 oral bioavailability and in vivo efficacy with favorable safety profiles.

## Introduction

The androgen receptor (AR) is the primary transcription factor driving prostate tumorigenesis and disease progression, making it a central therapeutic target throughout most stages of prostate cancer, including castration-resistant prostate cancer (CRPC). Although several second-generation AR pathway inhibitors (ARPIs) are available for CRPC treatment, a major challenge is the limited duration of clinical benefit due to primary or acquired resistance, which ultimately leads to mortality [1,2].

Resistance to ARPIs often results from restored AR signaling. The emergence of recurrent point mutations and constitutively active AR splice variants (AR-Vs) poses a major challenge to conventional therapies and is associated with poor prognosis [3]. Recurrent AR mutations, including those that convert antagonists into agonists, have been consistently detected, even after treatment with apalutamide and darolutamide [[2], [3], [4], [5]]. AR-Vs, particularly the frequently detected AR-V7, lack the ligand-binding domain (LBD) and thus evade current AR antagonists, yet they play a critical role in mediating cross-resistance among second-generation ARPIs [[6], [7], [8], [9]]. Although AR-V7 targeting has shown preclinical promise [[10], [11], [12]], clinical development of inhibitors such as masofaniten [13] and ONCT-534 [14] has not been successful.

Proteolysis-targeting chimeras (PROTACs) have emerged as a revolutionary approach in drug discovery, offering advantages such as overcoming resistance, targeting undruggable proteins, maintaining sustained effects, and enabling lower doses [15]. These bifunctional molecules induce proximity between target proteins and E3 ubiquitin ligases, facilitating proteasome-dependent degradation of the targets. Over twenty AR-targeting PROTACs have been reported, and several are in clinical trials [16]. While most target the LBD, AR-Vs have also been shown to be degradable. We and others have developed PROTACs that target AR via the N-terminal domain (NTD) [17,18] or DNA-binding domain [19,20], allowing degradation of both full-length AR and AR-Vs.

Despite these advances, orally bioavailable PROTACs remain a key goal. Our von Hippel-Lindau-based AR-NTD degrader, ITRI-90 [17], showed oral bioavailability with acceptable pharmacokinetics (PK), but further improvement was needed. Here, we report ITRI-148, which incorporates a Cereblon (CRBN) E3 ligase binding ligand and a rigid linker to enhance PK and antitumor potency. ITRI-148 achieves sustained AR signaling suppression and prevents AR-V7–driven reactivation, representing a promising candidate for advanced prostate cancer therapy.

## Materials and methods

### Chemistry

#### General experiment and information

Starting materials, reagents and solvents were purchased from commercial suppliers (Sigma- Aldrich, Acros, TCI, Alfa, Combi-Blocks, Matrix and Fischer) and were used as received without further purification. ^1^H spectra were obtained on Varian AS500 500 NMR-spectrometer in the indicated solvents. Chemical shifts are expressed in ppm (δ units) relative to TMS signal as internal standard. Flash column chromatography was performed on column packed with Merck silica gel 60 (0.063-0.200 μm). Preparative HPLC was carried out on a Jasco with a UV-975 detector (Inertsil ODS-3 column 30×250 mm, 5 μm reverse phase column, eluting CH_3_CN / H_2_O with 0.1 % TFA or without TFA, flow rate 42 mL / min, UV254 nm). Mass spectra with electronic impact (MS) were recorded from Micromass Quattro triple quadrupole mass spectrometer (Waters, USA). Solvents were reagent grade and, when necessary, they were purified and dried by standard methods. Concentration of the reaction solutions involved the use of rotary evaporator at reduced pressure.

#### Scheme of compound synthesis

The structures of all final compounds were verified by ^1^H-NMR, ^13^C-NMR, and high-resolution mass spectrometry (Fig. S1-S3). Purity of all final compounds was determined by analytical HPLC and listed in the compound description below. All final compounds reached >95 % purity (Fig. S4).

### Cell lines

CWR22Rv1, VCaP, DU145, HEK293T, LNCaP, and PC3 were obtained from ATCC; PNT2 from Sigma-Aldrich. LNCaP95 was provided by Dr. Jun Luo (Johns Hopkins University) and cultured in phenol red-free RPMI-1640 with 10 % charcoal-stripped FBS. C4-2B and C4-2B/MDVR were gifts from Dr. Allen C. Gao (UC Davis); C4-2B/MDVR was maintained with 10 μM enzalutamide. All lines were cultured per standard recommendations.

### Plasmids

pLenti4/TO/Flag-AR and pLenti4/TO/Flag-AR(∆LBD) plasmids are as described previously [17]. To generate pLenti4/TO/Flag-AR-V7 plasmid, the DNA fragment of AR-V7 was amplified from pEGFP-C1-AR V7, a gift from Michael A Mancini (Addgene plasmid # 86856) [21], and inserted into the pLenti4/TO plasmid carrying a N-terminal Flag tag between BamH1 and XhoI. The AR(L702H) and AR(H875Y) mutants were generated by site-directed mutagenesis of pCMV-Myc-AR, which is previously constructed and described in [22]. Primers used in the cloning and mutagenesis are:

AR-V7-f, CGCGGATCCAGAAGTGCAGTTAGGGCTGGG;

AR-V7-r, CCGCTCGAGTCAGGGTCTGGTCATTTTGAGA;

AR-L702H-f, CTCCTTTGCAGCCTTGCACTCTAGCCTCAATGAAC;

AR-L702H-r, GTTCATTGAGGCTAGAGTGCAAGGCTGCAAAGGAG;

AR-F875Y-f, CTATTGCGAGAGAGCTGTATCAGTTCACTTTTGACC;

AR-F875Y-r, GGTCAAAAGTGAACTGATACAGCTCTCTCGCAATAG.

Lentivirus carrying control pLKO.1 or shRNA vector targeting AR (TRCN0000003718) was produced following standard protocol.

### Immunoblotting

Cell lysis, Western blotting, and AR ubiquitination were performed as described in [17]. Primary antibodies: AR (Millipore 06-680), AR-V7 (RevMAb Biosciences 31-1109-00), PSA (ABclonal A4355), NKX3-1 (Cell Signaling Technology 92998), GAPDH (Cell Signaling Technology 2118) and β-Actin (Sigma-Aldrich A5441).

### Immunofluorescence and Proximity ligation assay (PLA)

Cells were seeded on coverslips 1 day prior to drug treatment, fixed with 4 % paraformaldehyde, and stained using anti-AR 441 (Santa Cruz Biotechnology sc-7305) following a standard protocol. PLA was conducted using Duolink® In Situ Detection Kit (Sigma-Aldrich) per manufacturer’s instructions. Antibodies: anti-Flag M2 and anti-CRBN (Sigma F3165, ZRB1304). DAPI was used for nuclear staining.

### Quantitative RT-PCR

Total RNA was extracted by TRIzol™ Reagent and subjected to cDNA synthesis using iScript™ Kit (Bio-Rad). Gene expression level was quantified by Bio-Rad CFX Real-Time PCR detection system using iTaq Universal SYBR Green Supermix. The primers used are as described in [17]. All samples were tested in triplicate, and the expression levels were normalized against *RPL13A* and *GAPDH*.

### RNA-sequencing

Total RNA quality and quantity were assessed using SimpliNano™ spectrophotometers and Qsep 100 analyzers. RNA sequencing libraries were prepared using the KAPA mRNA HyperPrep Kit, following the manufacturer’s protocol. mRNA was isolated using oligo-dT magnetic beads, fragmented, and converted into cDNA. After adapter ligation and fragment size selection (300–400 bp), libraries were amplified using KAPA HiFi HotStart ReadyMix, ensuring strand specificity. Library quality was verified using Qubit® 2.0 and an Agilent Bioanalyzer 2100 before sequencing on an Illumina NovaSeq X platform, generating 150 bp paired-end reads. Raw sequencing data were processed using CASAVA, and quality checks were performed with FastQC and MultiQC. Trimmomatic was used to remove adapters and low-quality reads, yielding high-quality "clean reads." These reads were mapped to the GRCh38 reference genome using HISAT2, and gene-level counts were obtained with featureCounts. Normalization and differential gene expression analysis was conducted using DESeq2. Enrichment analyses were performed using clusterProfiler for Gene Ontology (GO) and KEGG pathway analysis. Gene Set Enrichment Analysis (GSEA) was applied to identify biological functions and pathways from the molecular signatures database (MSigDB).

### Proteomics

Cell pellets were lysed in 0.1 % SDS, and proteins were diluted in triethylammonium bicarbonate (TEABC). Proteins were reduced with tris(2-carboxyethyl) phosphine (TCEP) and alkylated with methyl methylthiosulfonate (MMTS) as described [23]. Trypsin digestion (50:1 protein: trypsin, 25 μg: 0.5 μg) was performed overnight at 37°C. Tryptic peptides were SpeedVac dried and resuspended in 100 mM TEABC before tandem mass tag (TMT) 6-plex labeling (Thermo Fisher Scientific). Labeled peptides were pooled and analyzed by 2D LC-MS/MS using an Orbitrap Fusion Lumos Tribrid mass spectrometer. MS/MS spectra were searched against the Swiss-Prot human database using Mascot (v2.2.04) and processed with Proteome Discoverer (v1.4), including TMT reporter ion quantification. Parent and fragment ion tolerances were set at 10 ppm and 0.03 Da. Up to two missed tryptic cleavages were allowed. Variable modifications included methionine oxidation, N-terminal acetylation, and N-terminal glutamine pyroglutamination; cysteine methylthiolation was a fixed modification. TMT labeling was applied to lysine residues and peptide N-termini. Peptide identification required high-confidence matches, ≥6 amino acids, and <1 % false discovery rate.

### Cell viability

Cells were seeded in 96-well plates and treated with drugs the next day. After 7 days, viability was assessed using MTT assay.

### Pharmacokinetic analysis

All animal experiments followed NIH guidelines (NIH publication no 85–23, revised 1996) and were approved by ITRI IACUC (Approval nos. ITRI-IACUC-2020-001, −2020-014, and −2021-010).

Six to eight-week-old male BALB/c (BALB/cAnNCrlBltw) mice (22-30 g) were purchased from BioLASCO Taiwan Co., Ilan, Taiwan. *In vivo* PK analysis of ITRI-148 was performed with 10 mg/kg for oral (PO, *n* = 3) dosing route. Blood samples from the mice were collected using Microvette® CB 300 EDTA blood collection tubes by a lancet at 0.167 h (IV only), 0.5, 1, 2, 4, 7 and 24 h time points. The plasma was then subjected to LC-MS analysis to quantitate the drug concentration using calibration curves that were established with drug standards prepared in mice plasma.

### Xenografts studies

Immunodifficient male CB-17 SCID mice (CB17/Icr-*Prkdc^scid^*/IcrIcoCrlBltw) and NOD SCID mice (NOD.CB17-*Prkdc^scid^*/NCrCrlBltw) mice at four to six weeks of age were purchased from BioLASCO Taiwan Co., Ilan, Taiwan. All mice were housed in conventional cages in the AAALAC Full Accreditation (2011) in Industrial Technology Research Institute and were allowed to acclimatize and recover from shipping-related stress for one week prior to the study. The health of the mice was monitored by daily observations. Animals were kept in rooms at temperature of 22–26°C with 40–70 % humidity, positive pressure, 60 % air recirculation, ventilation rate 15–20 changes per hour, and a controlled light-dark cycle (12–12 hours). The order and location of each animal cage was randomly assigned to minimize potential confounders. CWR22Rv1 cells (5 × 10^6^) were suspended in 100 μL of PBS with 30 % Matrigel and subcutaneously implanted into the right flank of male CB-17 SCID mice. Tumors were measured with calipers, and tumor size was calculated as follows: tumor volume (V) = (*L* × S^2^)/2 (L, longest diameter, mm; S, shortest diameter, mm). When mean tumor volume reached 150–200 mm^3^, the mice were randomly assigned into indicated groups (*n* = 5-6/ group) and the day of initiating treatments was assigned as day 0. VCaP cells (5 × 10⁶) suspended in 50 % Matrigel were subcutaneously implanted into male NOD SCID mice. Once tumors reached approximately 250 mm³, castration surgery was performed. After tumor regression and subsequent regrowth to around 250 mm³, treatment with ITRI-148 was initiated. Oral (PO) gavage was used for the drug administration, including vehicle control and ITRI-148 treatments (7.5 mg/kg, 15 mg/kg, 30 mg/kg, or 60 mg/kg, BID). Tumor size and body weight of the animals were monitored and recorded two to three times per week. Mice were sacrificed when one of the tumor reached over 1000 mm^3^ in size. Tumors were collected and immediately subjected to liquid nitrogen freezing and homogenization. The homogenized tumor samples were stored at −80°C for subsequent western blotting analysis. The drug antitumor efficacy was presented as percentages of TGI of each tested animal, calculated as follows: [1 − (final tumor volume ^treated group^ – initial tumor volume ^treated group^) / (final tumor volume ^vehicle group^ – initial tumor volume ^vehicle group^)] × 100. Body weight of each mouse was also compared to that on the first day of treatment (day 0) and expressed as a percentage of day 0 value. All data points were included for calculation and the values are presented as mean ± SEM. Statistical analysis of the differences between two drug and vehicle treated groups was determined by two-tailed Student’s t-test.

### *In vitro* stability in plasma

The stability of ITRI-148 was assessed in plasma across four species: humans, dogs, rats, and mice. ITRI-148 added to pre-warmed plasma at 1 μM was maintained at the designated temperature, and aliquots were collected at 0, 0.5, 1, 2, and 4 hours after incubation. At each time point, 20 μL of the samples was taken to terminate the reaction, followed by LC-MS/MS analysis to determine the remaining compound concentration. Data from duplicated experiments was calculated and presented as mean ± SD.

### Metabolic stability in liver microsomes

The metabolic stability of all PROTAC compounds in the screening phase (Table 1) was evaluated using mouse liver microsomes. Incubations were performed at 37 °C in phosphate buffer (pH 7.4) containing NADPH (1 mM), 0.5 mg/mL microsomal protein and 1 μM ITRI-148 (0.25 % DMSO) for 0, 5, 20, or 30 minutes, followed by quenching with acetonitrile (1:3, v/v) for HPLC analysis. To evaluate the species-specific metabolism of ITRI-148, its stability was assessed in liver microsomes from human, dog, rat, and mouse using same incubation conditions as above, with the time extending to 0, 15, 30, 45, and 60 minutes, followed by acetonitrile quenching (1:10) and LC-MS/MS analysis.

**Table 1:** Assessment of the PROTAC compounds harboring different linkers.

Compound	Linker	Cell viability (%)	Microsome stability (% remaining)	PK (PO, 10 mg/kg)	
				AUC (h⋅ng/mL)	BA (%)
ITRI-148		1.71	97.2	9,901.5 ± 187.3	27.1
ITRI-157		1.64	98.5	298.9 ± 58.7	3.6
ITRI-160		10.76	93.8	201.3 ± 88.9	0.7
ITRI-165		40.12	96.4	2,252.9 ± 450.3	2.3

CWR22Rv1 cell viability was examined after 3 days of 10 μM compound treatment. Mice microsomal ability was detected by % compound remaining after 30 min of incubation. PO, oral administration; AUC, area-under-the-curve between 0 and 24 h; BA, bioavailability.

## Statistical analysis

All in vitro experiments were performed in triplicate and repeated at least three times. Two-tailed Student’s t-test was used for comparison; *p* < 0.05 was considered significant.

## Results

### Development of ITRI-148 for AR-NTD targeting

**Fig. 1:**
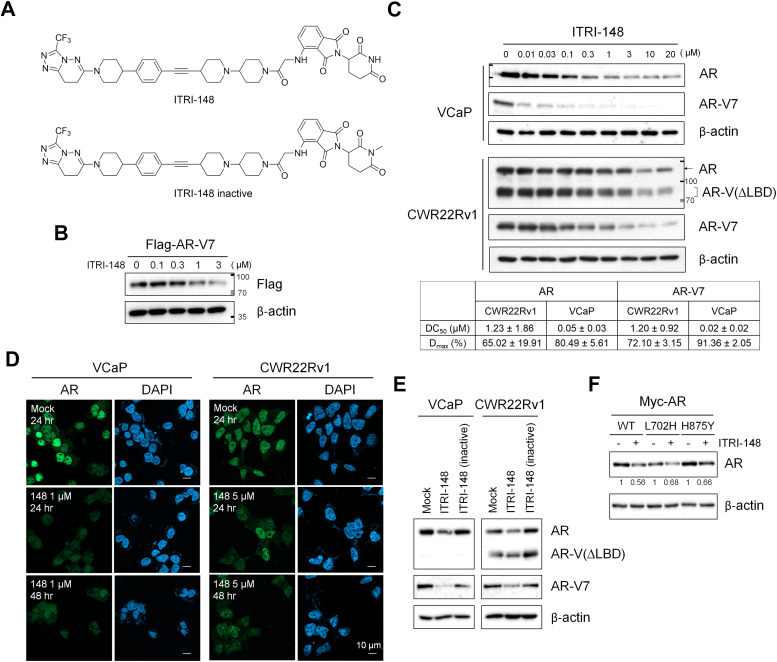
ITRI-148 induces degradation of both AR and AR-V7 proteins in CRPC cells. (A) Chemical structures of ITRI-148 and its inactive analog. (B) Expression of Flag-tagged AR-V7 in 293T cells following ITRI-148 treatment. Cells transiently expressing Flag-AR-V7 were pretreated with 50 μg/mL cycloheximide for 1 hour, then exposed to ITRI-148 at the indicated concentrations for 8 hours. AR-V7 expression was detected using an anti-Flag antibody. (C) Representative immunoblots showing AR protein levels in VCaP and CWR22Rv1 cells after 48 hours of ITRI-148 treatment. An N-terminal AR antibody was used to detect both full-length AR and LBD-truncated variants (AR-V(ΔLBD)) in CWR22Rv1 cells, while AR-V7 was detected using an AR-V7-specific antibody. DC₅₀ values were calculated from normalized band intensities, and Dₘₐₓ represents the maximal degradation achieved at 10 μM ITRI-148. (D) Immunofluorescence staining of AR after 24 or 48 hours of ITRI-148 treatment, detected using an N-terminal AR antibody. (E) Western blot analysis of AR and AR-V7 expression in cells treated with DMSO (mock), 10 μM ITRI-148, or the inactive analog. (F) Western blot analysis of ectopically expressed wild-type (WT) and mutant Myc-tagged AR in DU145 cells following 24 hours of treatment with 10 μM ITRI-148. Quantified AR levels are as indicated.

We previously reported a series of AR-NTD-targeting PROTACs constructed with the core structure of AZD3514 serving as the AR-binding moiety and linkers based on polyethylene glycol [17]. Among them, ITRI-126 is a CRBN-dependent degrader utilizing lenalidomide as the E3 ligase binding moiety. Although ITRI-126 exhibited substantial AR-degrading efficiency in cells, its PK profile was suboptimal. To improve the *in vivo* efficacy, we tested rigidified linkers in combination with thalidomide. Compounds achieving at least 60 % AR-V7 degradation after 24 hours of treatment were further assessed for their cell-inhibiting ability, microsomal stability, and oral PK profiles (Table 1). ITRI-148, which incorporates a piperidine and alkyne motif (Fig. 1A), demonstrated superior performance across all evaluated aspects.

To confirm the targeting of ITRI-148 on AR-NTD, 293T cells transiently expressing Flag-tagged AR-V7 were treated with the compound following a 1-hour pretreatment of cycloheximide to minimize the interference of newly synthesized proteins. After 8 hours of ITRI-148 treatment, the level of Flag-AR-V7 was significantly reduced, indicating a degradation event mediated by this compound (Fig. 1B). Additionally, we explored the druggability of the AR-NTD and performed pocket prediction using the DoGSiteScorer [24,25] platform on the full-length AR model generated by AlphaFold [26]. Among the identified cavities, Pocket 7 locating on the NTD within residues 221–320, the AR fragment employed in our original ligand screen [17], exhibited a high druggability score (drugScore = 0.86) (Fig. S5A). On the other hand, Pocket 6 overlaps with the known DHT-binding site within AR-LBD [27], also exhibited a high drugScore (0.86). The identification of Pocket 7 thus suggested a promising site for small molecule intervention beyond the conventional LBD.

We next performed molecular docking of ITRI-148 and AZD3514 to AR. The docking results demonstrated comparable binding affinities, with ITRI-148 showing a best pose of −8.199 kcal/mol and AZD3514 at −7.467 kcal/mol within Pocket 7 (Fig. S5B). Notably, pose clustering and RMSD distribution indicated stable and specific ligand accommodation in the cavity. AZD-3514 formed hydrophobic interactions with I232, A236, K237, and L257, and hydrogen bonds with S233 and S258. ITRI-148 engaged hydrophobic interactions with I232, Q235, L239, and L254, and formed hydrogen bonds with R13, L239, and E261, as well as a halogen bond with K237. These findings indicate that both ligands interact with key residues within the AR-NTD, particularly I232 and K237, suggesting that engagement with these residues may contribute to their pharmacodynamic effects (Fig. S5C). Taken together, these findings provide structural support for targeting the AR-NTD pocket in drug discovery efforts, and validate the rational design of ITRI-148 as a promising AR degrader targeting NTD.

### ITRI-148 degrades AR and AR-V7 via the proteasome-dependent system

The ability of ITRI-148 degrading endogenous AR proteins was next tested in VCaP and CWR22Rv1 cells, both of which are known to express high levels of full-length AR and LBD-deleted AR variants, including AR-V7, AR-V9 and AR-V567es [28,29]. Using an AR N-terminal antibody, ITRI-148 was shown to effectively decrease the expression of all active AR species. The reduction of AR-V7 variant was confirmed using an AR-V7–specific antibody (Fig. 1C). Compared to ITRI-126, ITRI-148 exhibited stronger AR degrading potency in VCaP cells, with half-maximal degradation concentration (DC_50_) for AR and AR-V7 ranging in 50 nM and 20 nM, respectively (Fig. 1C table, [17]). In CWR22Rv1 cells, the DC_50_ values were higher, within the low micromolar range. The maximum degradation level (D_max_) achieved by ITRI-148 in VCaP cells was 80 % for AR and 91 % for AR-V7. We found that CWR22Rv1 cells express lower level of CRBN compared with VCaP and other AR-positive prostate cancer cell lines, which may account for the higher DC₅₀ observed in CWR22Rv1 (Fig. S6A). Immunofluorescence staining using an N-terminal antibody confirmed that AR expression decreased within 24 hours of ITRI-148 treatment and reached a stronger extent after 48 hours (Fig. 1D). Importantly, an inactive analog of ITRI-148 derived from N-methylated thalidomide, known to prevent the glutarimide-dependent interaction with CRBN [30,31], failed to degrade the AR proteins, indicating that CRBN E3-ligase activity is essential for ITRI-148–mediated AR degradation (Fig. 1A, 1E). In addition to full-length AR and LBD-deleted AR variants, ITRI-148 also degraded ectopically expressed Myc-tagged AR mutants L702H and H875Y in DU145 cells, two of the most commonly recurrent ARPI-resistant mutations found in metastatic CRPC patients (Fig. 1F) [32,33], reinforcing the ability of ITRI-148 to target all active forms of AR.

**Fig. 2:**
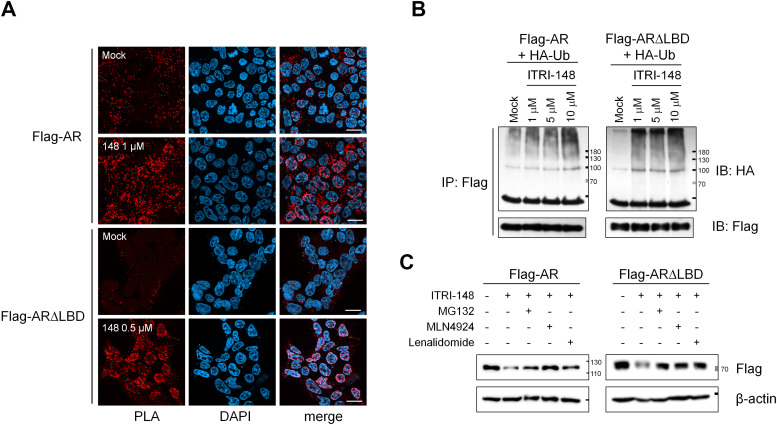
ITRI-148 induces AR degradation through a ubiquitin-proteasome-dependent mechanism. (A) Proximity ligation assay (PLA) detecting the physical interaction between AR and CRBN upon ITRI-148 treatment. 293T cells transiently expressing Flag-AR or Flag-AR∆LBD were treated with DMSO or ITRI-148 for 8 hours, followed by PLA detection using anti-Flag and anti-CRBN antibodies. (B) Ubiquitination of AR and AR∆LBD proteins. 293T cells transfected with Flag-AR or Flag-AR∆LBD and HA-Ub were incubated with ITRI-148 and MG132. AR proteins were immunoprecipitated using anti-Flag, and ubiquitination of the AR proteins was detected by western blotting with anti-HA. (C) Western blot of Flag-AR and Flag-AR∆LBD in DU145 cells treated with ITRI-148 alone or in combination with MG132 (5 μM), MLN4924 (0.5 mM), or lenalidomide (80 μM). Inhibitors were added for 8 or 10 hours after the 24-hour ITRI-148 treatment. AR expression was detected using anti-Flag antibody.

To further demonstrate that ITRI-148 induces CRBN recruitment, proximity ligation assays (PLA) were performed in 293T cells expressing Flag-AR or Flag-ARΔLBD (LBD-deleted AR). Within 8 hours of ITRI-148 treatment, proximity between AR proteins and endogenous CRBN was detected (Fig. 2A). While PLA signals for full-length AR were observed in both the nucleus and cytosol, ARΔLBD signals were enriched in the nucleus. In 293T cells co-expressing Flag-AR or Flag-ARΔLBD with HA-ubiquitin, ITRI-148 induced a dose-dependent increase in AR ubiquitination (Fig. 2B). As the ubiquitination level of an unrelated Flag-tagged protein, PDK1, remained unchanged, this result confirmed that the ITRI-148-mediated ubiquitination event was specific to AR (Fig. S6B). Co-treatment with proteasome inhibitor MG132, NEDD-8 inhibitor MLN4924, or excess lenalidomide blocked the degradation of Flag-AR and Flag-ARΔLBD in DU145 cells, further substantiating the action of ITRI-148 through a ubiquitin-proteasome-dependent mechanism (Fig. 2C).

### ITRI-148 effectively targets AR signaling

**Fig. 3:**
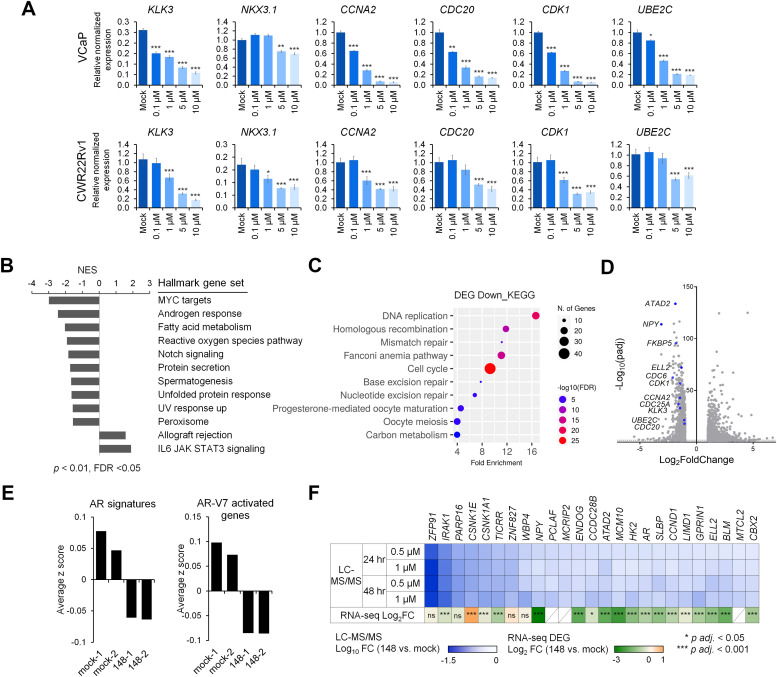
ITRI-148 specifically targets the AR pathway. (A) qRT-PCR analysis of AR and AR-V7 target gene expression in VCaP and CWR22Rv1 cells after 48-hour drug treatment. *GAPDH* and *RPL13A* served as reference genes. Statistical significance: *p < 0.05, **p < 0.01, ***p < 0.001. (B-D) RNA-seq analysis of VCaP cells treated with either mock or 1 μM ITRI-148 for 48 hours. (B) GSEA analysis and NES (normalized enrichment score) of top hallmark gene sets enriched in ITRI-148-treated cells. (C) KEGG pathway analysis of significantly downregulated DEGs (differentially expressed genes, fold change (FC) > 2, padj < 0.05). (D) Volcano plot of genes significantly altered in drug-treated cells. Known AR target genes are marked in blue. Dotted line indicates *p* = 0.01. (E) AR and AR-V7 signature scores were calculated by summing the z-scores of individual gene expression values. (F) LC-MS/MS analysis of altered protein abundance upon ITRI-148 treatment, alongside corresponding transcriptomic changes from RNA-seq. Protein and mRNA fold changes compared against mock treatment are shown as Log_10_ FC and Log_2_ FC, respectively. Asterisks denote adjusted p-values; ns = not significant.

The transcriptional impact of ITRI-148 in VCaP and CWR22Rv1 cells was examined by qRT-PCR analysis of AR and AR-V7 target genes. *KLK3* and *NKX3-1* are well-established AR targets, whereas *CCNA2, CDC20, CDK1*, and *UBE2C* are reported to be activated by AR-V7, although they can also be regulated by AR-FL and other AR-Vs in a complex, context-dependent manner [34]. This context dependence was evident in CWR22Rv1 and VCaP cells: in CWR22Rv1 cells, expression of *CCNA2, CDC20, CDK1*, and *UBE2C* was not suppressed by enzalutamide, whereas in VCaP cells, these genes appeared dependent on AR-FL activity, consistent with the known role of AR in cell cycle regulation (Fig. S7A). Importantly, all the above-mentioned genes were significantly downregulated in response to increasing ITRI-148 doses (Fig. 3A, S7A), confirming the effective suppression of both AR and AR-V activities in these cells. Furthermore, in androgen-responsive cell models such as LNCaP, C4-2B, and VCaP, stimulation with 1 nM DHT following ITRI-148 treatment failed to boost *KLK3* expression, indicating robust inhibition of AR signaling by ITRI-148 (Fig. S7B).

RNA-seq analysis of ITRI-148-treated VCaP cells also confirmed these transcriptomic changes. Gene set enrichment analysis (GSEA) revealed significant downregulation of androgen response genes, and AR-regulated pathways including MYC targets [35] and fatty acid metabolism [36] (Fig. 3B). Additionally, pathways related to cell cycle regulation, homologous recombination, DNA replication, and repair were among the top 10 downregulated pathways in degrader-treated cells (Fig. 3C). Suppression of AR- and AR-V7-regulated gene signatures [37,38] further substantiated the effective AR targeting by ITRI-148 (Fig. 3D, 3E).

To evaluate specificity, we performed proteomic analysis on VCaP cells treated with 0.5 μM or 1 μM ITRI-148 for 24 and 48 hours. AR was among the top 25 proteins consistently and significantly decreased across all treatment groups. Several AR target proteins, including ATAD2, HK2, and Cyclin D1, were also significantly reduced (Fig. 3F). Many of these downregulated proteins showed corresponding reductions at their transcript level, indicating a result of AR-dependent transcriptional suppression. Additionally, strong decline in immunomodulatory drug (IMiD) neosubstrates such as ZFP91, CSNK1E, CSNK1A1, and ZNF827 [39,40] occurred without corresponding transcript reduction. This non-specific protein degradation likely results from the thalidomide moiety in ITRI-148.

### ITRI-148 potently inhibits the viability of CRPC and enzalutamide-resistant cells

**Fig. 4:**
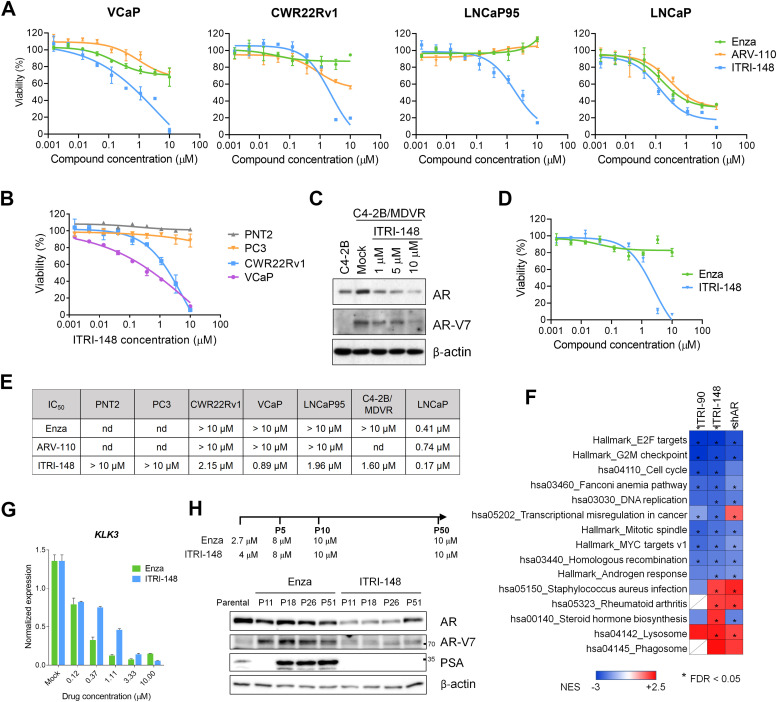
ITRI-148 inhibits viability of AR-V7-expressing CRPC cells and provides durable AR suppression. (A) MTT assay measuring cell viability after 7 days of treatment with enzalutamide (Enza), ARV-110, or ITRI-148. (B) Anti-proliferative effect of ITRI-148 in normal prostate epithelial vs. CRPC cell lines. (C) Western blot analysis of AR and AR-V7 in C4-2B and C4-2B/MDVR cells after 48-hour ITRI-148 treatment. (D) MTT assay of C4-2B/MDVR cells following 7-day Enza or ITRI-148 treatment. (E) IC_50_ values for each drug/cell line (A, B, D) are summarized in the table; nd = not determined. (F) GSEA of significantly enriched gene sets in C4-2B/MDVR cells treated with AR PROTACs (ITRI-90, ITRI-148) for 48 hours or AR shRNA (shAR) for 72 hours. DEGs were determined relative to DMSO or pLKO.1 vector control. NES values for top pathways are displayed in a heatmap. (G) *KLK3* expression in C4-2B cells after 48-hour treatment with Enza or ITRI-148. *RPL13A* was used for normalization. (H) Comparison of AR suppression by Enza vs. ITRI-148 long-term treatment in C4-2B cells. AR, AR-V7, and PSA expression in the drug-treated cells were detected across treatment timepoints.

To evaluate the anti-proliferative potency of ITRI-148, we compared it with enzalutamide and Arvinas ARV-110, an AR-LBD targeting PROTAC [41]. CRPC cell lines expressing AR-V7: VCaP, CWR22Rv1, and LNCaP95, along with hormone-dependent LNCaP cells, were tested. While ITRI-148 showed comparable growth-inhibiting potency to the other two drugs in LNCaP cells, it exhibited significantly greater efficacy in CRPC cells (Fig. 4A). Normal prostate epithelial cells (PNT2) and AR-negative CRPC cells (PC3) were insensitive to ITRI-148 (Fig. 4B), highlighting potent and selective inhibition of cancer cell viability through AR targeting. On the other hand, in an acquired enzalutamide-resistant CRPC model, C4-2B/MDVR, which expresses high levels of AR and AR-V7 compared to parental C4-2B cells [42], ITRI-148 effectively degraded AR and suppressed viability (Fig. 4C–4E). Transcriptomic analysis of C4-2B/MDVR cells treated with ITRI-148, ITRI-90, or an AR-knockdown construct revealed that both degraders closely mimicked the effects of AR knockdown, further confirming target specificity (Fig. 4F).

### ITRI-148 exhibits a sustained effect of AR inhibition

Clinical and preclinical studies have shown that AR variant induction, particularly AR-V7, is a key mechanism underlying resistance to ARPIs [1]. To compare the long-term effects of different AR-inhibiting modalities, we subjected C4-2B cells to extended treatments with enzalutamide or ITRI-148. Initial short-term testing in C4-2B cells revealed comparable levels of *KLK3* suppression at low micromolar concentrations (Fig. 4G). To generate drug-adapted lines, C4-2B cells were treated with doses achieving ∼90 % *KLK3* suppression. Surviving cells were expanded and maintained under increasing drug concentrations up to 10 μM, then cultured in 10 μM drug for an additional forty passages. Throughout the experiment, cells were collected for western blot analysis to monitor AR, AR-V7, and PSA (encoded by *KLK3*) expression. AR-V7 expression emerged after 11 passages in the enzalutamide-treated group, while it remained undetectable in ITRI-148-treated cells; AR expression also stayed low (Fig. 4H). PSA was initially suppressed in the enzalutamide group but rebounded following AR-V7 induction. In contrast, ITRI-148-treated cells showed no PSA re-expression, suggesting durable AR signaling suppression.

*AKR1C3*, which encodes a steroidogenic enzyme essential for the synthesis of potent androgens, is an AR-repressed gene [43,44] whose upregulation promotes the survival of CRPC and ARPI-resistant cells [45,46]. In cells exposed to enzalutamide, *AKR1C3* expression was strongly induced during the early phase and subsequently decreased alongside the re-expression of *KLK3* during drug adaptation (Fig. S8, Fig. 4G). By contrast, although *AKR1C3* was also induced in ITRI-148 treated cells, its expression remained at a moderate level throughout the experimental period, in parallel with persistently low *KLK3* expression (Fig. S8). These findings support the notion that ITRI-148 is capable of sustaining AR signaling suppression and suggest that AKR1C3 may contribute to resistance mechanisms against AR degraders.

### ITRI-148 is orally bioavailable and exhibits potent antitumor activity

**Fig. 5:**
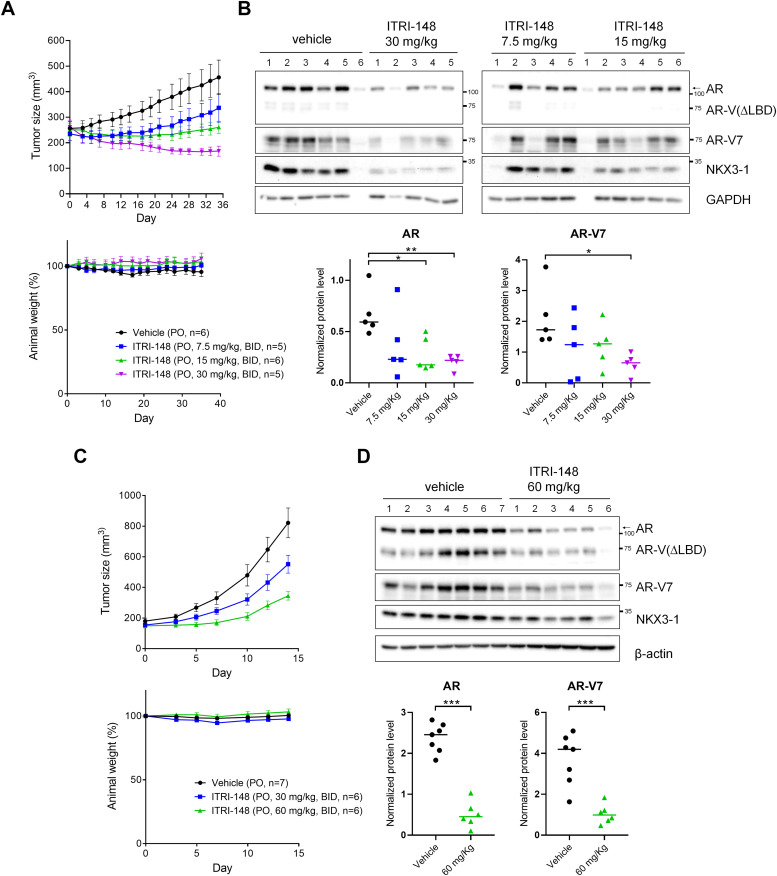
ITRI-148 suppresses tumor growth in VCaP-castrated and CWR22Rv1 xenograft models. (A, B) Castrated SCID mice bearing VCaP xenografts were treated with ITRI-148 orally at 7.5, 15, or 30 mg/kg BID. (C,D) Hormone-intact mice with CWR22Rv1 xenografts received 30 or 60 mg/kg ITRI-148 BID orally. (A, C) Tumor volume and body weight were monitored over the treatment course. (B, D) Western blot analysis of AR, AR-V7 and NKX3-1 in tumors harvested at study endpoint; normalized quantification of AR and AR-V7 is shown below. One sample each from the VCaP-vehicle (#6) and VCaP-15 mg/kg (#1) groups lacked detectable GAPDH and was excluded. *p < 0.05, **p < 0.01, ***p < 0.001.

Comparing to ITRI-126, ITRI-148 demonstrated a significantly improved oral PK profile, with increased AUC/Dose and enhanced bioavailability (Table 2). Antitumor efficacy of orally administered ITRI-148 was evaluated in castrated VCaP xenografts and hormone-intact CWR22Rv1 xenograft models. In castrated mice bearing VCaP xenografts, treatment with 7.5 mg/kg or 15 mg/kg twice daily (BID) resulted in tumor growth inhibition (TGI) to 49.06 % and 95.08 %, respectively. At 30 mg/kg BID, ITRI-148 substantially regressed tumors, with an average TGI of 147.07 %. (Fig. 5A). AR and AR-V7 expression in the tumors was reduced in a dose-dependent manner (Fig. 5B). In CWR22Rv1 xenografts, oral dosing at 30 mg/kg and 60 mg/kg BID significantly reduced tumor size and AR/AR-V7 levels, with TGI of 38.08 % and 69.39 %, respectively (Fig. 5C, 5D). Importantly, the expression of AR target NKX3-1 was correspondingly decreased in the tumors upon ITRI-148 treatment, confirming the suppression of AR signaling *in vivo* (Fig. 5B, 5D). No toxicity reflected by weight-loss of the animals was observed.

**Table 2:** Pharmacokinetic profiles of ITRI-148 and ITRI-126 following oral administration.

	ITRI-148 (PO, 10 mg/kg)	ITRI-126 (PO, 10 mg/kg)
T_max_ (h)	4.0 ± 0.0	0.7 ± 0.2
C_max_ (ng/mL)	732.7 ± 45.0	95.7 ± 47.1
AUC_last_ (h⋅ng/mL)	9,901.5 ± 187.3	203.7 ± 85.9
AUC/Dose (h⋅kg⋅ng/mL/mg)	990.1	20.4 ± 8.6
BA (%)	27.1 ± 0.5	4.6 ± 1.9

T_max_, the time takes to reach C_max_; C_max_, maximum drug concentration; AUC_last_, area-under-the-curve between 0 and 24 h; AUC/Dose, area-under-the-curve between 0 and 24 h per dosing; BA, bioavailability.

**Fig. 6:**
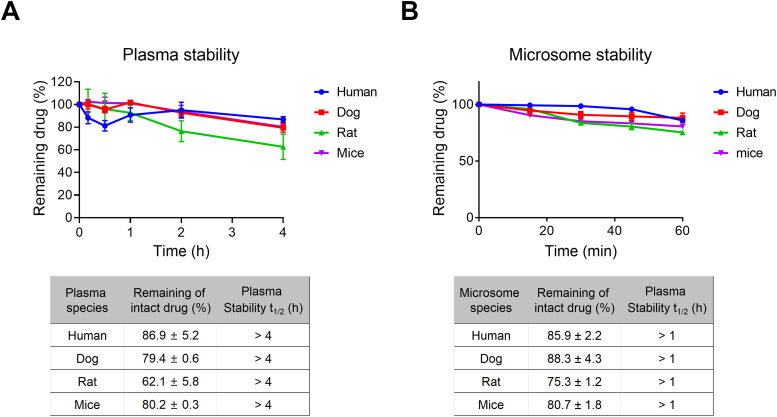
ITRI-148 shows favorable plasma and metabolic stability in vitro across species. (A) Residual ITRI-148 levels in plasma from human, dog, rat, and mouse after incubation for the indicated times (*n* = 2). (B) Microsomal stability of ITRI-148 in the same four species over time.

Moreover, *in vitro* stability assays showed that ITRI-148 had a plasma half-life exceeding four hours in all species tested (Fig. 6A). The compound also demonstrated significant stability in liver microsomes from humans, dogs, rats, and mice (Fig. 6B), indicating phase I metabolism via hepatic CYP450 enzymes is not the primary route of elimination. These data confirm that ITRI-148 possesses a favorable PK and stability profile, supporting its oral bioavailability and efficacy against enzalutamide-resistant CRPC tumors.

### Toxicity profiles of ITRI-148 in rats

To further assess drug safety, a repeated-dose toxicity study and toxicokinetic (TK) profiling were conducted in rats following oral administration of ITRI-148 (Fig. S9A). Compared with single-dose administration, the TK profile showed a dose-dependent increase in plasma drug concentrations after 7 repeated doses, without evidence of saturation at the highest tested dose (1000 mg/kg) (Fig. S9B). Specifically, in the 100 mg/kg group, systemic exposure after repeated dosing was comparable to that observed after a single dose, whereas the 300, 600, and 1000 mg/kg groups exhibited progressively higher C_max_ and AUC values, with the most pronounced increase observed at 1000 mg/kg. These TK results supported the selection of 1000 mg/kg as the maximum dose for the rat toxicity study, corresponding to more than a 10-fold margin over the efficacious dose used in our xenograft model. The TK profile also confirmed sufficient systemic exposure for toxicological evaluation.

In the repeated-dose toxicity study, no mortality occurred in any dose group during the 7-day dosing period or the subsequent 7-day recovery phase. Body weight and food consumption remained within normal variation across all groups (Fig. S9C). Hematological parameters measured on Days 7 and 14 were within normal reference ranges for all animals. Minor and reversible changes were observed in neutrophil, lymphocyte, and monocyte percentages (Table S1). Similarly, serum biochemical markers, including AST, ALT, BUN, and creatinine, showed no significant increases relative to controls, indicating no apparent hepatic or renal toxicity at any tested dose (Table S2). On Day 7, organ weight analysis revealed significant decreases in thymus and spleen weights in the 1000 mg/kg group compared with vehicle controls, but this phenomenon was fully reversed during the recovery phase. No dose-dependent changes were observed in other organs (Table S3).

Collectively, these findings demonstrate that ITRI-148 exhibited no apparent systemic toxicity in rats. Consistent with the absent signs of toxicity in mice indicated by animal weight loss, these results support the overall safety of ITRI-148 for further development.

## Discussion

Linker optimization plays a critical role in PROTAC performance. Rigid linkers are known to enhance solubility, cell permeability, PK properties, stabilization of the target-E3 ligase ternary complex, and oral absorption [[47], [48], [49], [50]]. In this study, we introduce ITRI-148, an AR-NTD PROTAC featuring a piperidine-alkyne linker and a thalidomide E3 ligase ligand. Druggability prediction and molecular docking of ITRI-148 and AZD3514 with AR provide structural insights supporting direct targeting of the AR-NTD by these compounds. In vitro assays demonstrate that ITRI-148 effectively induces potent degradation of all active forms of AR, including full-length AR, LBD-truncated AR-Vs such as AR-V7, and recurrent mutants such as L702H and H875Y, underscoring its potential of overcoming resistance acquired to conventional AR antagonists. ITRI-148 acts through the ubiquitin-proteasome system, rapidly recruiting both full-length and LBD-truncated AR to CRBN in the nucleus within 8 hours, enabling efficient degradation of constitutively active AR-Vs and ligand-bound AR. Degradation of ligand-bound AR is supported by the observation that ITRI-148 efficiently degrades full-length AR in cells cultured in hormone-containing medium, as all *in vitro* experiments were conducted without hormone deprivation.

Incorporation of a rigidified linker in ITRI-148 also significantly improved its PK properties. At the oral doses estimated to exceed the *in vitro* IC₅₀ in VCaP or CWR22Rv1 cells, marked tumor shrinkage or suppression was observed in castrated VCaP and hormone-intact CWR22Rv1 xenografts. As androgen levels significantly impact AR stability and the efficacy of conventional antagonists [51,52], the ability of ITRI-148 to degrade ligand-bound AR highlights NTD-targeting as a potentially efficient therapeutic modality, regardless of hormone fluctuations in patients.

Transcriptomic and proteomic analyses confirmed that ITRI-148 selectively disrupts AR signaling by promoting its degradation and the consequent transcriptional suppression. In the enzalutamide-resistant C4-2B/MDVR model, transcriptomic changes induced by ITRI-148 closely mirrored those observed following AR knockdown, particularly affecting cell cycle, mitotic, and checkpoint-related genes. Similar to AR, which is well-established to be essential for cell cycle regulation in prostate cancer cells, AR-V7 promotes castration- and ARPI-resistance by driving cell cycle progression [[53], [54], [55]]. ITRI-148′s inhibition of these pathways highlights its ability to counteract AR-V7-mediated therapeutic escape.

Remarkably, ITRI-148 outperformed both enzalutamide and ARV-110 in suppressing the viability of multiple CRPC models, including VCaP, CWR22Rv1, LNCaP95, and C4-2B/MDVR, further validating the advantages of AR-NTD degraders over LBD-targeting agents. Although ITRI-148 also induces degradation of known IMiD neosubstrates, likely due to its unmodified thalidomide moiety, future chemical modifications may enhance substrate selectivity and minimize off-target effects. Despite the potential off-target activity associated with the unmodified thalidomide, the repeated-dose toxicity study and TK profiling of ITRI-148 in rats indicate an overall favorable safety profile for this compound. In support of translational safety, it is demonstrated that ITRI-148 is well tolerated at up to 1000 mg/kg/day—exceeding >10-fold the efficacious dose used in xenograft models. Observed histological and hematological alterations were mild and fully reversible upon cessation of dosing. These findings establish a favorable preclinical safety margin and reinforce the compound’s suitability for further development.

A notable strength of ITRI-148 is its prolonged inhibition of AR signaling. While both ITRI-148 and enzalutamide initially suppress AR activity, enzalutamide treatment ultimately results in AR-V7 upregulation and PSA re-expression. In contrast, ITRI-148 sustains AR degradation without inducing AR-V7, suggesting its potential to prevent therapy-induced resistance. Although a slight increase in AR expression was observed following long-term ITRI-148 treatment, possibly indicating emerging resistance to its AR-degrading activity, an important consideration is the mechanism by which a subset of cells survives prolonged treatment despite suppression of AR signaling. Given that AKR1C3 upregulation has been implicated as a resistance mechanism to ARPI therapy through its roles in AR coactivation and stabilization [56], it is plausible that, in the presence of residual AR proteins, elevated AKR1C3 expression may represent a general mechanism conferring resistance to AR-PROTACs. Investigations into AR-independent pathways, such as glucocorticoid receptor activation or neuroendocrine transdifferentiation [2], are also warranted to better understand potential long-term implications of AR-NTD PROTAC therapy in clinical settings.

## Conclusions

ITRI-148 is a CRBN-based AR-NTD PROTAC featuring a rigid linker that significantly enhances oral PK properties and antitumor efficacy in models of castration-resistant and enzalutamide-resistant prostate cancer. Through degradation of both AR and AR-V7, ITRI-148 provides durable suppression of AR signaling, underscoring AR-NTD-targeted degradation as a promising therapeutic strategy for advanced prostate cancer. In addition, its demonstrated stability and favorable safety profile further support the potential of ITRI-148 in future drug development.

## Data availability

RNA-seq data have been deposited in the NCBI Gene Expression Omnibus under accession number GSE297577 and are publicly available as of the date of publication. Further inquiries can be directed to the corresponding authors.

## Funding

This work is supported by National Science and Technology Council, Taiwan (NSTC 113-2320-B-182-005-), and Ministry of Economic Affairs, Taiwan (MOEA 112-EC-17-A-22-1651).

## Declaration of Generative AI and AI-assisted technologies in the writing process

During the preparation of this work the author(s) used ChatGPT in order to improve the language. After using this tool/service, the author(s) reviewed and edited the content as needed and take(s) full responsibility for the content of the publication.

## CRediT authorship contribution statement

**Chiu-Lien Hung:** Writing – original draft, Validation, Supervision, Methodology, Funding acquisition, Formal analysis, Data curation, Conceptualization. **Wen-Ning Hsu:** Investigation. **Tsan-Chun Wang:** Investigation. **Wan-Ru Chen:** Visualization, Investigation. **Yu-Ting Chen:** Investigation, Formal analysis. **Zong-Keng Kuo:** Investigation. **Tsan-Lin Hu:** Investigation. **Yu-Chin Lin:** Investigation. **Hsun-Hao Yeh:** Investigation. **Han-Chen Lin:** Investigation. **Chia-Jung Yu:** Investigation, Formal analysis. **Chih-Wei Fu:** Resources. **Hao-Hsuan Liu:** Resources. **Hung-Chih Hsu:** Resources. **Po-Hung Lin:** Resources. **See-Tong Pang:** Resources. **Chih-Ho Lai:** Resources. **Ling-Yu Wang:** Writing – review & editing, Writing – original draft, Visualization, Validation, Supervision, Methodology, Investigation, Funding acquisition, Formal analysis, Data curation, Conceptualization.

## Declaration of competing interest

The authors declare the following financial interests/personal relationships which may be considered as potential competing interests: Chiu-Lien Hung, Tsan-Lin Hu, Yu-Chin Lin, Chih-Wei Fu, Hao-Hsuan Liu has patent #US 12,152,035 B2 licensed to Industrial Technology Research Institute, Hsinchu, Taiwan. Other authors declare that they have no known competing financial interests or personal relationships that could have appeared to influence the work reported in this paper.

## Appendix Supplementary materials

Image, application 1
