# Supplementary material for: Oral bioavailable ITRI-148 degrades androgen receptor variants and overcomes antiandrogen resistance in advanced prostate cancer
Source: Neoplasia. 2025 Nov 13;71:101253. doi: 10.1016/j.neo.2025.101253 (PMC12661400; doi:10.1016/j.neo.2025.101253)
Supplement: Supplementary file 1 [file mmc1.pdf]

Supplementary Materials for:

Oral Bioavailable ITRI-148 Degrades Androgen Receptor Variants and Overcomes Antiandrogen Resistance in Advanced Prostate Cancer

Chiu-Lien Hung[1], Wen-Ning Hsu[2], Tsan-Chun Wang[3], Wan-Ru Chen[1], Yu-Ting Chen[1], Zong-Keng Kuo[1], Tsan-Lin Hu[1], Yu-Chin Lin[1], Hsun-Hao Yeh[3], Han-Chen Lin[3], Chia-Jung Yu[3,4,5,6], Chih-Wei Fu[1], Hao-Hsuan Liu[1], Hung-Chih Hsu[7,8], Po-Hung Lin[8,9], See-Tong Pang[8,9], Chih-Ho Lai[3,10], Ling-Yu Wang[2,3,7,*]

[1] Department of Preclinical Drug Discovery Technology, Biomedical Technology and Devices Research Labs, Industrial Technology Research Institute, Hsinchu 31040, Taiwan.
[2] Department of Biochemistry and Molecular Biology, Chang Gung University, Taoyuan 33302, Taiwan.
[3] Graduate Institute of Biomedical Sciences, Chang Gung University, Taoyuan 33302, Taiwan.
[4] Department of Cell and Molecular Biology, Chang Gung University, Taoyuan 33302, Taiwan.
[5] Molecular Medicine Research Center, Chang Gung University, Taoyuan 33305, Taiwan.
[6] Department of Thoracic Medicine, Chang Gung Memorial Hospital at Linkou, Taoyuan 33305, Taiwan.
[7] Division of Hematology-Oncology, Chang Gung Memorial Hospital at Linkou, Taoyuan 33305, Taiwan.
[8] School of Medicine, College of Medicine, Chang Gung University, Taoyuan 33305, Taiwan.
[9] Division of Urology, Department of Surgery, Chang Gung Memorial Hospital at Linkou, Taoyuan 33305, Taiwan.
[10] Department of Microbiology and Immunology, Chang Gung University, Taoyuan 33302, Taiwan.

*Corresponding author contact: lywang@mail.cgu.edu.tw
Department of Biochemistry and Molecular Biology, Chang Gung University
No.259, Wenhua 1st Rd., Guishan District.,
Taoyuan City 33302, Taiwan
Phone: +886-3-211-8800 ext.3989
Fax: +886-3-211-8042

Materials and Methods

General procedure for synthesis of ITRI-148 & ITRI-148- inactive

148-P2(1eq.) and Methyl-148-C (1eq.) was dissolved in DMF, then Et_3N(5eq.), Propylphosphonic anhydride solution 50% in DMF (1.5eq.) was added dropwise to the reaction at 0°C. The mixture was stirred at 0°C for 2 h. The reaction was diluted with DCM and washed with water. The aqueous layer was then extracted (3xDCM). The combined organics layers were washed with water and dried ($MgSO_4$) before being concentrated in vacuo. The crude was then purified using Flash column chromatography on silica gel using a gradient of 0 to 10% of MeOH in DCM to afford the yellow solid.

ITRI-148

Yield = 58.6%. ^{1}H-NMR (500 MHz, DMSO-d_6): δ 11.08(s,1H), 7.59 (t, J = 7.5 Hz , 1H), 7.29 (d, J = 7.5 Hz, 2H), 7.21 (d, J = 8 Hz,2H), 7.11-7.04 (m, 3H),5.05 (dd, J = 5.5, 12.5 Hz, 1H), 4.40 (d, J = 11.5 Hz, 1H), 4.26 (s, 1H), 4.20–4.12 (m, 3H), 3.91(d, J = 12.5 Hz, 1H), 3.13 (t, J = 7.5Hz, 2H), 2.93–2.88(m, 6H) , 2.84–2.82 (m, 1H) , 2.73–2.72 (m, 3H) , 2.65–2.56 (m, 4H) , 2.31 (s, 2H), 2.03-2.01 (m, 1H), 1.81-1.74 (m, 6H) , 1.64-1.55 (m, 4H) , 1.48-1.44 (m, 1H), 1.34-1.28 (m, 1H). ^{13}C-NMR (125 MHz, DMSO-d6), δ(ppm):173.30, 170.56, 169.24, 167.80, 166.36, 160.81, 146.61, 145.87, 145.84, 139.45, 139.14, 136.62, 132.44, 131.80, 127.44, 121.40, 120.08, 118.74, 117.95, 111.22, 109.91, 93.36, 81.51, 61.66, 49.01, 47.93,44.94, 44.05,43.67, 41.70, 32.40, 31.44, 28.22, 27.76, 22.60, 20.27, 17.01. ESI-MS m/z: found [M + H]+ =853.3790; Purity = 98.3%. Chemical Formula: $C_{44}H_{47}F_3N_{10}O_5$.

ITRI-148- inactive

Yield = 79.0%. ^{1}H-NMR (500 MHz, DMSO-d_6): δ 7.60 (t, J = 7.5 Hz , 1H), 7.29 (d, J = 8.5 Hz, 2H), 7.21 (d, J = 8.5 Hz,2H), 7.11-7.05 (m, 3H),5.12 (dd, J = 5.5, 12.5 Hz, 1H), 4.40 (d, J = 12 Hz, 1H), 4.27 (s, 1H), 4.20–4.11 (m, 3H), 3.91(d, J = 12.5 Hz, 1H), 3.13 (t, J = 8Hz, 2H), 3.01 (s, 3H), 2.99–2.90(m, 6H) , 2.85–2.80 (m, 1H) , 2.76–2.73 (m, 3H) , 2.65–2.57 (m, 4H) , 2.31 (s, 2H), 2.05-2.03 (m, 1H), 1.81-1.74 (m, 6H) , 1.64-1.56 (m, 4H) , 1.54-1.44 (m, 1H), 1.31-1.22 (m, 1H). ^{13}C-NMR (125 MHz, DMSO-d6), δ(ppm):172.25, 170.27, 169.22, 167.77, 166.35, 160.80, 146.60, 145.87, 145.84, 139.45, 139.14, 136.64, 132.43, 131.78, 127.42, 121.42, 120.08, 118.76, 117.94, 111.22, 109.89, 93.43, 81.48, 61.60, 49.58, 47.94, 45.78, 44.04, 43.69, 41.70, 32.52, 31.58, 28.30, 27.82, 27.06, 21.82, 20.27, 17.00. ESI-MS m/z: found [M + H]+ =867.3935; Purity = 98.3%. Chemical Formula: $C_{45}H_{49}F_3N_{10}O_5$.

Plasmid and primers

Flag-PDK1 plasmid is as described previously [1]. Primers used for *AKR1C3* qRT-PCR are:
AKR1C3-f: GTCATCCGTATTTCAACCGGAG
AKR1C3-r: CCACCCATCGTTTGTCTCGT

Molecular Docking of ITRI-148 to AR NTD

Molecular docking was performed using AutoDock Vina (v1.2.7) [2] to investigate the potential binding of ITRI-148 to AR N-terminal domain (NTD). Due to the intrinsically disordered nature of the AR-NTD, no experimentally resolved full-length AR structure is available. Therefore, a full-length 3D model of AR was generated using AlphaFold3 [3] based on the UniProt sequence (UniProt ID: P10275). While the NTD region exhibited low pLDDT scores, the overall model aligned well with the AR ligand-binding domain structure bound to the AR 20–30 peptide (PDB ID: 2Q7I). The 3D structure of ITRI-148 was built using ChemDraw, converted with Open Babel (v3.1.1), and minimized before docking. Putative binding pockets were predicted using DoGSiteScorer [4, 5], and a top-ranked pocket (simpleScore = 0.58; drugScore = 0.86) was selected based on its druggability and geometry. Docking simulations were run within a $30 \times 30 \times 30$ Å^3 grid centered at (X = 8.823, Y = 8.527, Z = −34.257). Protein preparation involved water removal, polar hydrogen addition, and Kollman charge assignment via AutoDock Tools (v1.5.7). The ligand was assigned Gasteiger charges. Docking was performed with exhaustiveness = 16, generating 10 poses. Binding conformations were visualized and analyzed in PyMOL (v2.6, Schrödinger Inc.).

Repeated-dose toxicity and toxicokinetic studies

Repeated-dose oral toxicity study and toxicokinetic (TK) profiling was conducted in Sprague-Dawley rats. ITRI-148 was administered once daily using oral gavage at doses of 100, 300, 600, and 1000 mg/kg/day for 7 consecutive days, followed by a 7-day recovery period. Each dose group comprised 3 animals designated for toxicokinetic (TK) analysis and 6 animals for dose-range finding (DRF) evaluation. Clinical observations, body weights, and food consumption were monitored daily throughout the dosing and recovery periods. After the first (Day 0) and last (Day 6) dosing, serial blood samples were collected at the pose-dose time points of 0.5, 1, 2, 4, 7, 24, 48 and 72 hours for TK profiling analysis. Plasma concentrations of ITRI-148 were quantified using a validated LC-MS/MS method. Analysis was performed using a Sciex QTrap 4000 mass spectrometer coupled with a Shimadzu LC system. The chromatographic separation was achieved on a C18 reverse-phase column with a gradient elution of acetonitrile and 0.1% formic acid in water. The lower limit of quantification (LLOQ)

was 5 ng/mL. All samples were analyzed in duplicate. Hematology and serum clinical chemistry analyses were performed on Day 7 (end of dosing) and Day 14 (end of recovery). At necropsy, organ weights (including heart, liver, kidney, spleen, thymus, and adrenal glands) were recorded, and gross pathological changes were examined. All procedures were conducted in compliance with institutional animal care and use guidelines.

References

[1] Wang LY, Hung CL, Chen YR, Yang JC, Wang J, Campbell M, Izumiya Y, Chen HW, Wang WC, Ann DK, et al. (2016). KDM4A Coactivates E2F1 to Regulate the PDK-Dependent Metabolic Switch between Mitochondrial Oxidation and Glycolysis *Cell Rep* **16**, 3016-3027.

[2] Trott O, Olson AJ (2010). AutoDock Vina: improving the speed and accuracy of docking with a new scoring function, efficient optimization, and multithreading *J Comput Chem* **31**, 455-461.

[3] Jumper J, Evans R, Pritzel A, Green T, Figurnov M, Ronneberger O, Tunyasuvunakool K, Bates R, Zidek A, Potapenko A, et al. (2021). Highly accurate protein structure prediction with AlphaFold *Nature* **596**, 583-589.

[4] Volkamer A, Griewel A, Grombacher T, Rarey M (2010). Analyzing the topology of active sites: on the prediction of pockets and subpockets *J Chem Inf Model* **50**, 2041-2052.

[5] Volkamer A, Kuhn D, Grombacher T, Rippmann F, Rarey M (2012). Combining global and local measures for structure-based druggability predictions *J Chem Inf Model* **52**, 360-372.

[6] Hung CL, Liu HH, Fu CW, Yeh HH, Hu TL, Kuo ZK, Lin YC, Jhang MR, Hwang CS, Hsu HC, et al. (2023). Targeting androgen receptor and the variants by an orally bioavailable Proteolysis Targeting Chimeras compound in castration resistant prostate cancer *EBioMedicine* **90**, 104500.

A

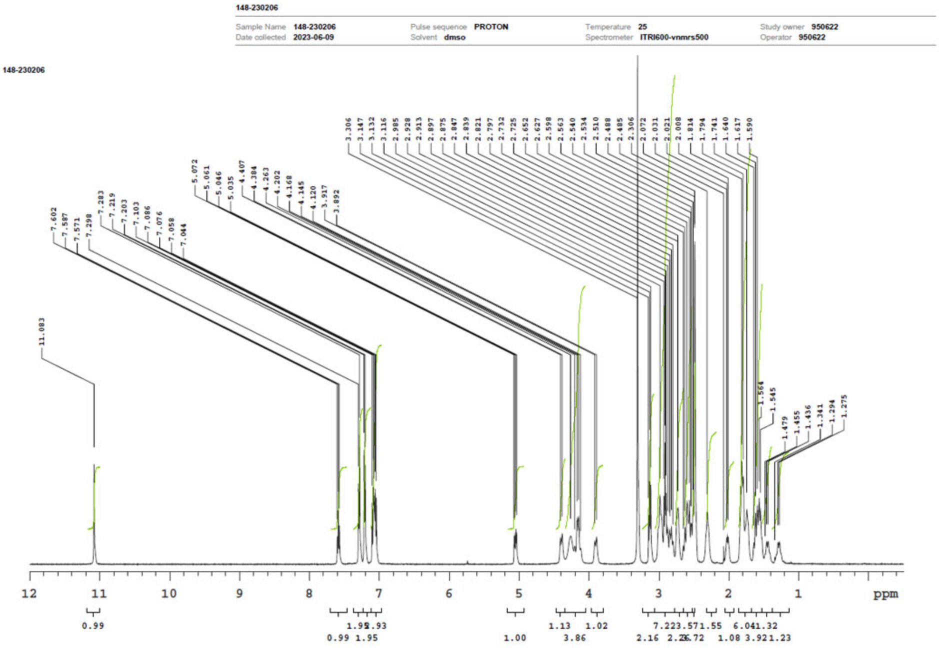

B

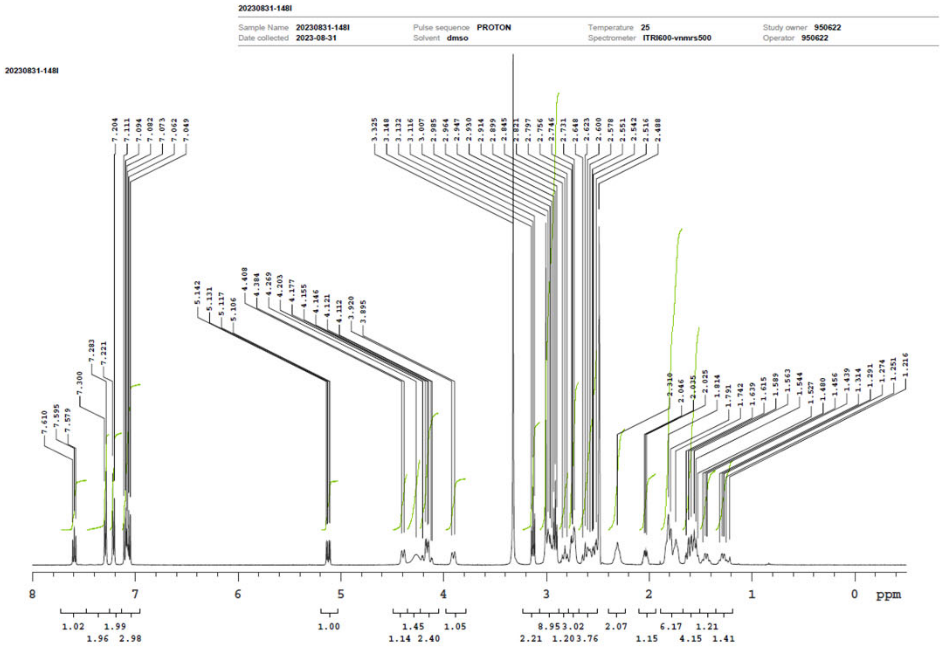

Fig. S1. ^{1}H-NMR spectra of (A) ITRI-148 and (B) ITRI-148 inactive.

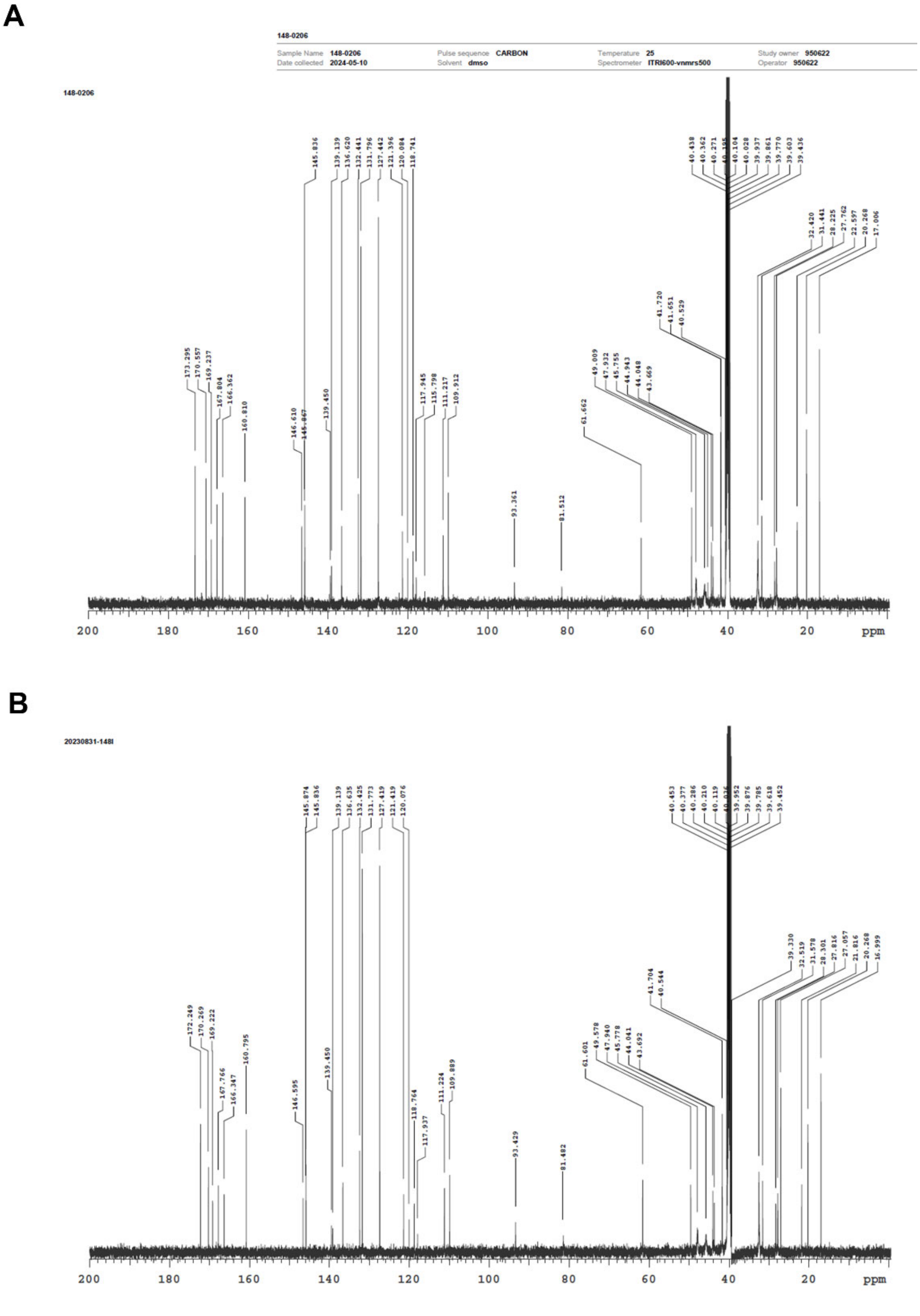

Fig. S2. ^{13}C-NMR spectra of (A) ITRI-148 and (B) ITRI-148 inactive.

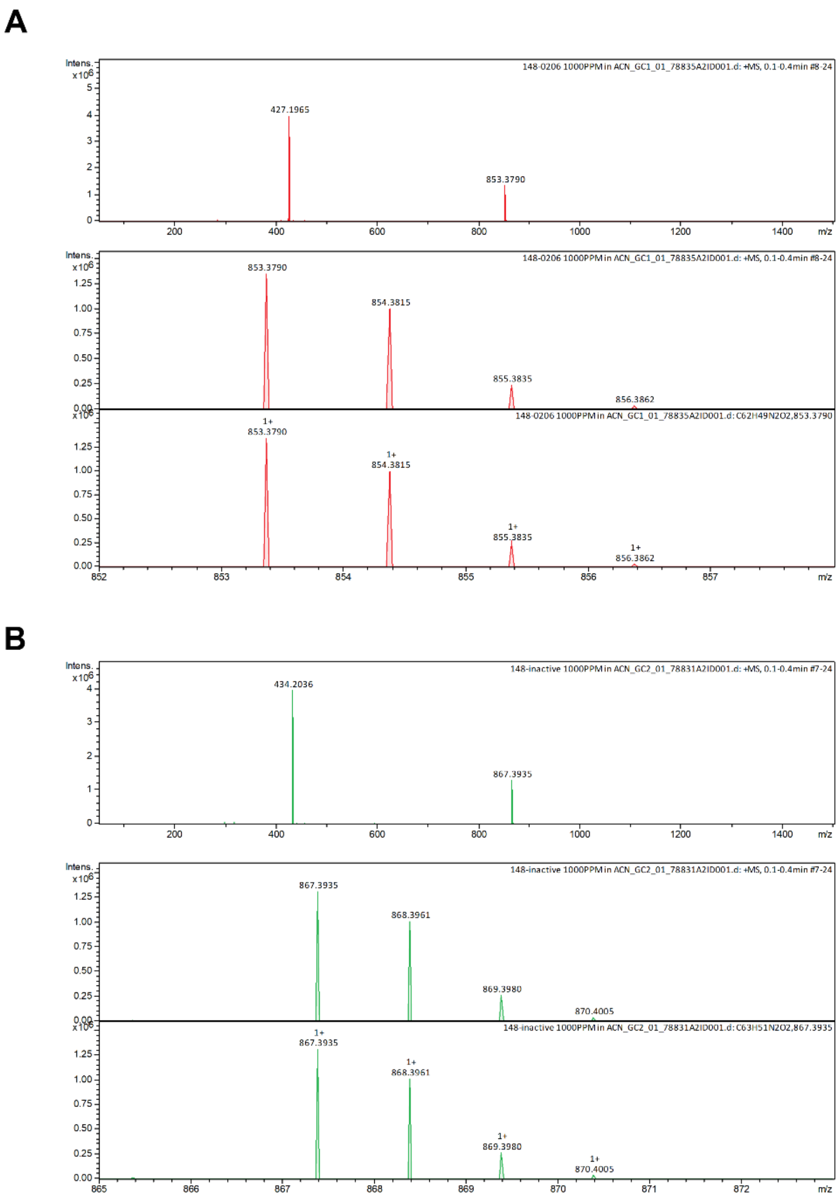

Fig. S3. HRMS spectra of (A) ITRI-148 and (B) ITRI-148 inactive.

A

Column:
Agilent Poroshell 120 EC-C18, 4.6*100mm, 2.7 µm
Mobile phase : A: 0.1% TFA in water; B: Acetonitrile/Methanol 3:1
Column Temp: 30 ℃
Wavelength: 244 mm

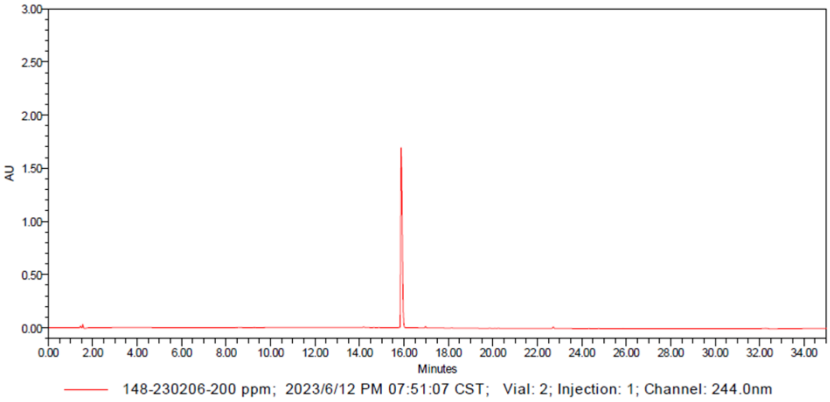

	RT	Area	Hight	% Area
1	14.187	15480	5000	0.19
2	14.88	4418	1683	0.05
3	15.779	8207	3293	0.1
4	15.876	8111891	1693865	98.33
5	16.969	42712	13936	0.52
6	17.167	2811	914	0.03
7	19.855	4122	1297	0.05
8	20.097	2092	739	0.03
9	22.719	55495	15022	0.67
10	24.766	2463	839	0.03

B

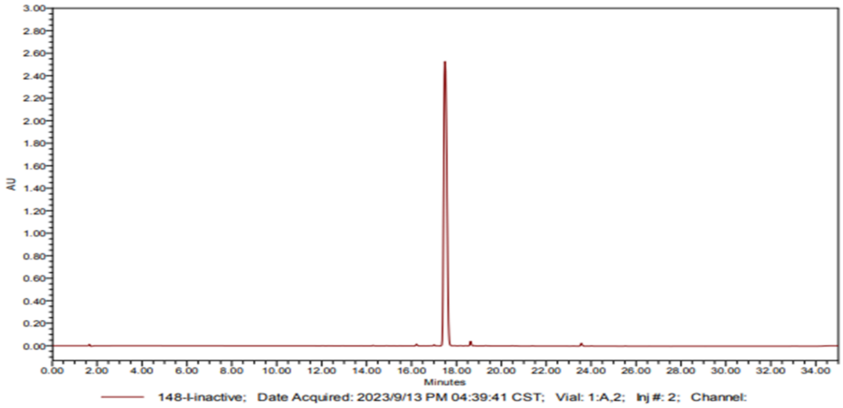

	RT	Area	Hight	% Area
1	14.288	21492	4903	0.08
2	16.223	62565	14899	0.25
3	18.633	151643	38222	0.6
4	19.312	12286	2895	0.05
5	20.492	12013	2745	0.05
6	17.494	24904344	2561718	98.33
7	21.379	9073	2125	0.04
8	23.567	119443	25586	0.47
9	17.011	34710	8863	0.14

Fig. S4. HPLC spectra of (A) ITRI-148 and (B) ITRI-148 inactive.

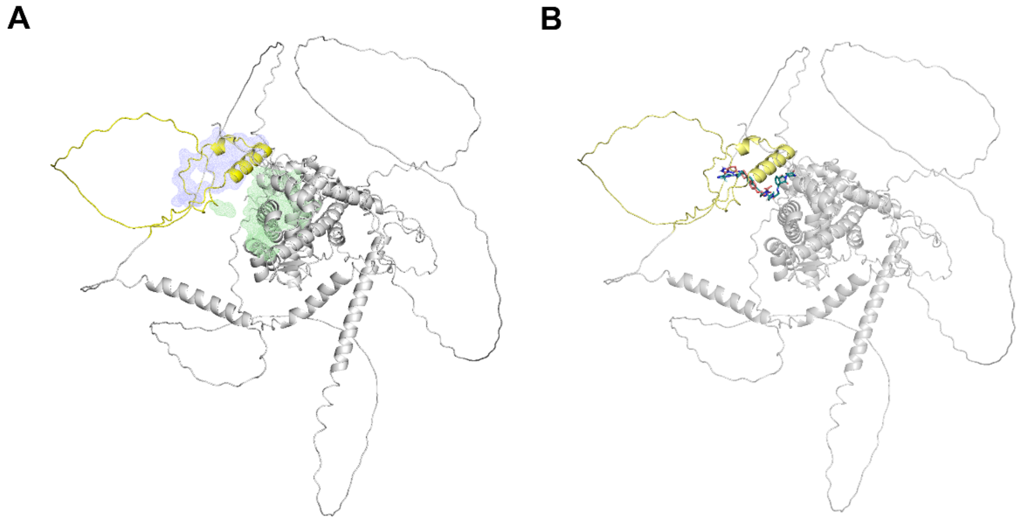

Pocket	Volume ($Å^3$)	Surface ($Å^2$)	depth	surf/vol	simpleScore	drugScore
6	842.65	1010.9	29.53	1.19967	0.57	0.86
7	764.16	1516.49	25.98	1.98452	0.58	0.86

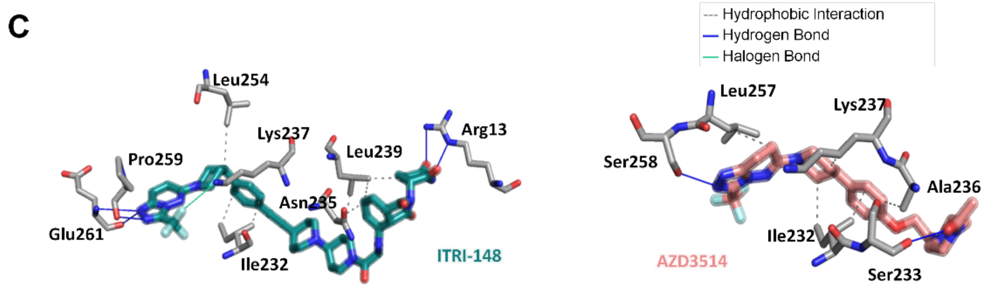

Fig. S5. Structural modeling and docking of AR with ITRI-148 and AZD3514.

(A) Predicted binding pockets of the full-length AR AlphaFold model identified by DoGSiteScorer. Pocket 6 (green) overlaps with the known DHT-binding site in the ligand-binding domain (LBD), while Pocket 7 (blue) is located in the N-terminal domain (NTD). Residues 221–320, previously used in warhead screening [6], are shown in yellow. (B, C) Docking poses of ITRI-148 (cyan, left) and AZD3514 (magenta, right) within Pocket 7 were generated using AutoDock Vina 2 with the AR-FL_AlphaFold model as a rigid receptor. (C) Enlarged views highlight key interactions between the ligands and AR residues. Both ITRI-148 and AZD3514 engage residues I232 and K237 within the NTD through hydrophobic and hydrogen-bonding interactions.

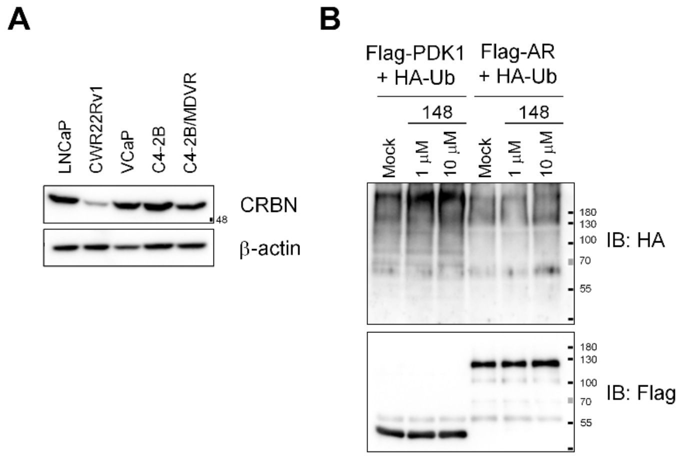

Fig. S6. Confirmation of CRBN expression and AR-specific ubiquitination.

(A) Western blot analysis of CRBN expression in the indicated prostate cancer cell lines. (B) ITRI-148 induced ubiquitination is specific to AR. 293T cells transfected with Flag-PDK1 or Flag-AR and HA-Ub were incubated with ITRI-148 and MG132. The Flag-tagged proteins were immunoprecipitated, and the ubiquitination levels were detected by western blot with a rabbit anti-HA antibody (BioLegend #Poly9023).

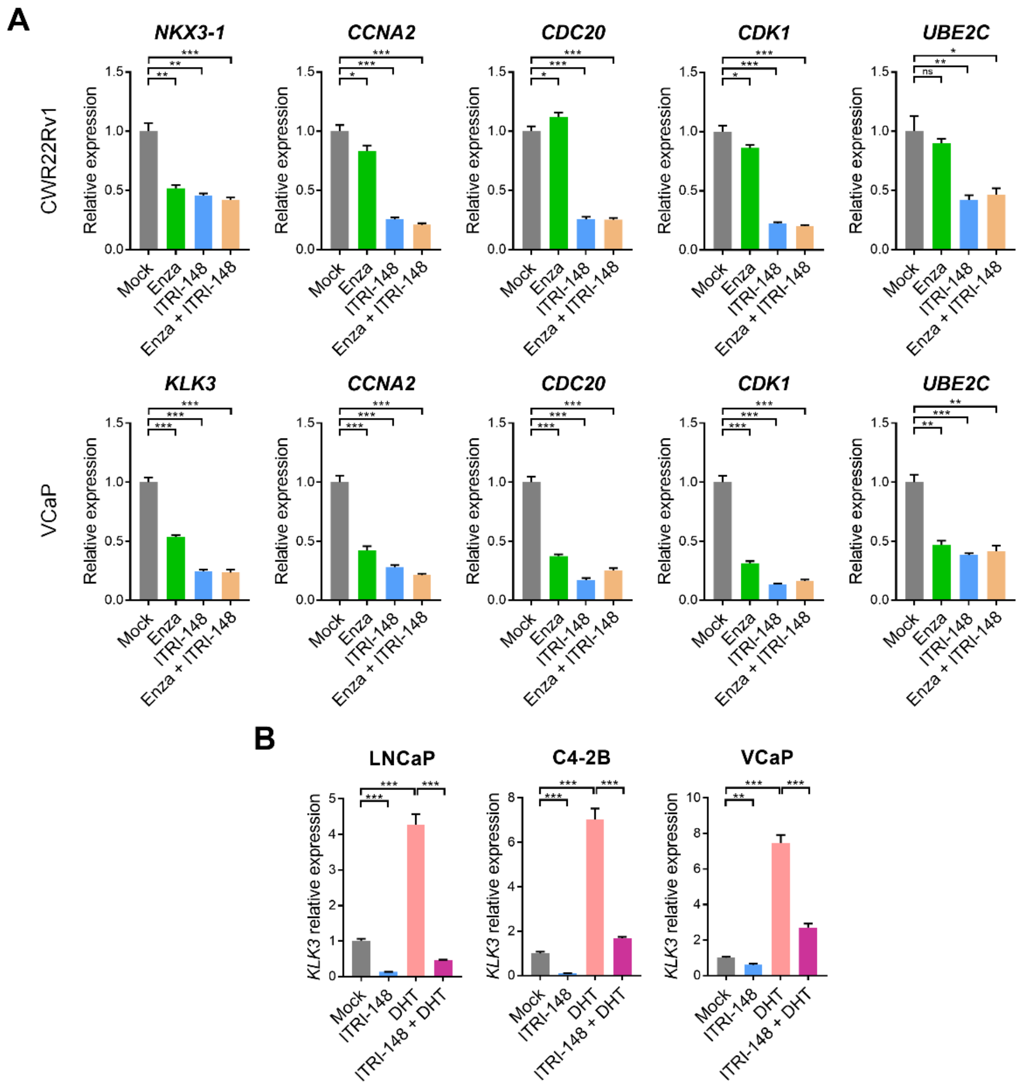

Fig. S7. Confirmation of AR signaling suppression.

(A) Literature-reported AR-V7 target genes are differentially regulated by AR-FL in CWR22Rv1 and VCaP cells. Cells treated with 10 μM enzalutamide (Enza), 5 μM ITRI-148 or both for 2 days were analyzed for their gene expression by qRT-PCR. *RPL13A* was used for normalization. (B) AR reactivation assay following ITRI-148 treatment. Cells were seeded in media containing 10% charcoal-dextran-treated FBS 1 day prior to drug treatment. 10 μM ITRI-148 or DMSO (mock) was added for 24 hours, followed by an additional 24-hour treatment with or without 1 nM DHT. *KLK3* expression was analyzed by qRT-PCR, with *RPL13A* used for normalization. Statistical significance: $*p < 0.05$, $**p < 0.01$, $***p < 0.001$.

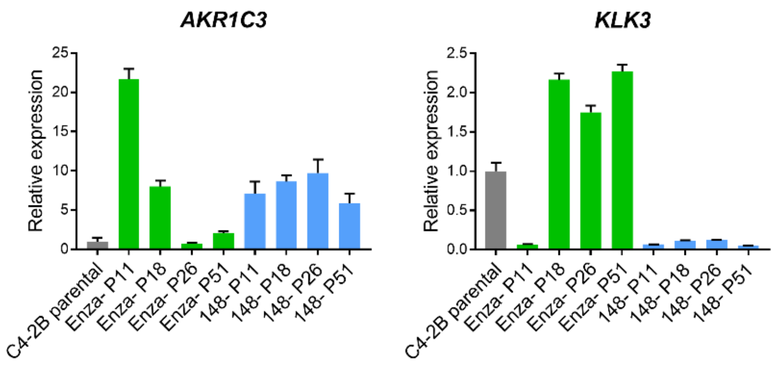

Fig. S8. Upregulation of *AKR1C3* in the long-term drug treated C4-2B cells conversely correlates with AR signaling.

AKR1C3 and *KLK3* expression in the drug-treated cells were detected by qRT-PCR across enzalutamide/ ITRI-148 treatment timepoints. *RPL13A* was used for normalization.

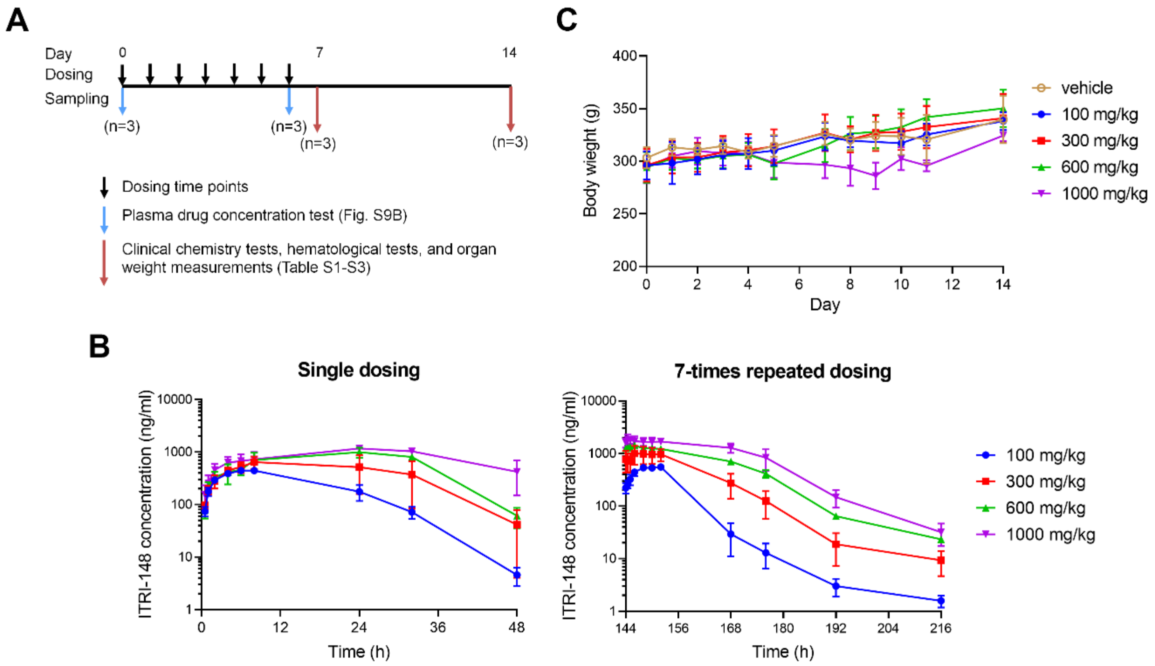

PK parameter	Single dosing				7-times repeated dosing			
	100 mg/kg	300 mg/kg	600 mg/kg	1000 mg/kg	100 mg/kg	300 mg/kg	600 mg/kg	1000 mg/kg
C_{max} (ng/mL)	465.7 ± 13.6	659.0 ± 183.6	914.0 ± 295.9	1,001.0 ± 303.4	561.3 ± 62.1	1,051.7 ± 335.0	1,438.3 ± 130.1	1,906.7 ± 361.3
AUC_{last} (h·ng/mL)	9,295.3 ± 956.8	20,145.9 ± 8,629.0	27,098.2 ± 10,160.4	40,366.6 ± 17,317.6	8,749.6 ± 1,218.2	20,508.1 ± 7,335.0	35,226.2 ± 1,560.4	56,029.3 ± 6,008.3
AUC_{24} (h·ng/mL)	7672.1 ± 500.1	12,977.9 ± 4,220.2	15,088.8 ± 4,600.0	17,430.4 ± 6,382.5	8,332.2 ± 1,091.3	17,041.1 ± 5,582.9	25,225.8 ± 1,550.0	36,619.2 ± 2,445.3

Fig. S9. Repeated-dose toxicity study of ITRI-148 in Sprague-Dawley rats.

(A) Schematic of the study design. Animals received oral ITRI-148 at 100, 300, 600, or 1000 mg/kg/day for 7 consecutive days, followed by a 7-day recovery period. (B) Plasma concentrations of ITRI-148 were measured in blood samples collected after single dosing (left) or 7-times repeated dosing (right). Data are expressed as mean ± SEM (n=3 per dose group in each time point). The pharmacokinetic (PK) parameters are summarized in the table below. C_{max}, maximum drug concentration; AUC_{last}, area-under-the-curve between 0 and last time point; AUC_{24}, area-under-the-curve between 0 and 24 h. (C) Absolute body weights were recorded prior to drug administration and throughout the study period. No animal exhibited >10% weight loss in any treatment group. Data are expressed as mean ± SEM (Day 0 to 7, n=9 per dose group; Day 8 to 14, n=3 per dose group).

Table S1. Hematological parameters in Sprague-Dawley rats after oral administration of ITRI-148

Day 7	Vehicle	ITRI-148			
		100 mg/kg	300 mg/kg	600 mg/kg	1000 mg/kg
WBC (x10^3 cells/µL)	13.51 ± 4.5	8.72 ± 2.37	8.19 ± 2.89	8.68 ± 4.58	7.48 ± 2.72
RBC (x10^6 cells/µL)	7.85 ± 0.2	7.51 ± 0.13	7.15 ± 0.4	7.51 ± 0.53	7.52 ± 0.31
HGB (g/dL)	22.1 ± 1.1	20.3 ± 0.2	20 ± 1.4	20.6 ± 1.9	20.7 ± 1.3
PLT (x10^3 cells/µL)	1209 ± 146	915 ± 368	956 ± 108	986 ± 252	892 ± 66*
Day 14	Vehicle	ITRI-148			
		100 mg/kg	300 mg/kg	600 mg/kg	1000 mg/kg
WBC (x10^3 cells/µL)	9.9 ± 4.86	7.52 ± 5.51	9.56 ± 1.57	5.27 ± 2.39	8.28 ± 0.31
RBC (x10^6 cells/µL)	8.43 ± 0.92	8.18 ± 1.17	8.91 ± 0.17	7.87 ± 0.45	8.33 ± 0.35
HGB (g/dL)	22.9 ± 1.8	21.9 ± 3.2	24.1 ± 0.4	21.6 ± 0.8	21.6 ± 1
PLT (x10^3 cells/µL)	1032 ± 117	1049 ± 231	1128 ± 236	1022 ± 446	1140 ± 222

Hematology parameters were measured at the end of dosing (Day 7) and after the recovery period (Day 14). Data are expressed as mean ± SEM (n = 3 per dose group per sampling day). All values were within normal reference ranges. Significant differences from vehicle controls are indicated with an asterisk.

Table S2. Clinical chemistry parameters in Sprague-Dawley rat serum after oral administration of ITRI-148

Day 7	Vehicle	ITRI-148			
		100 mg/kg	300 mg/kg	600 mg/kg	1000 mg/kg
AST (U/L)	124.3 ± 2.9	188.7 ± 59	177 ± 37.4	196 ± 66	132.7 ± 14.5
ALT (U/L)	63 ± 11.1	102.3 ± 40.6	85.3 ± 10	76.7 ± 2.1	68 ± 15.4
BUN (mg/dL)	18.37 ± 2.6	17.73 ± 2.8	20.53 ± 2.06	21.8 ± 1.81	16.77 ± 2.32
CRE (mg/dL)	0.57 ± 0.06	0.63 ± 0.06	0.63 ± 0.06	0.57 ± 0.06	0.57 ± 0.06
Day 14	Vehicle	ITRI-148			
		100 mg/kg	300 mg/kg	600 mg/kg	1000 mg/kg
AST (U/L)	166.7 ± 31.5	136.7 ± 10.4	122 ± 5.3	170 ± 41.7	150.3 ± 43
ALT (U/L)	104 ± 48.3	84.3 ± 31.6	60.7 ± 7.1	69.3 ± 11.5	94.3 ± 41.2
BUN (mg/dL)	16.97 ± 5.65	13.23 ± 1.12	10.9 ± 1.13	13.03 ± 0.83	16.23 ± 4.71
CRE (mg/dL)	0.5 ± 0.1	0.4 ± 0.1	0.33 ± 0.06	0.47 ± 0.06	0.37 ± 0.06

Parameters were measured at the end of dosing (Day 7) and after the recovery period (Day 14). Data are expressed as mean ± SEM (n = 3 per dose group per sampling day).

Table S3. Absolute organ weights in Sprague-Dawley rats after oral administration of ITRI-148

Day 7	Vehicle	ITRI-148			
		100 mg/kg	300 mg/kg	600 mg/kg	1000 mg/kg
Heart	1.26 ± 0.181	1.417 ± 0.443	1.369 ± 0.173	1.269 ± 0.209	1.259 ± 0.118
Thymus	0.286 ± 0.041	0.419 ± 0.079	0.377 ± 0.079	0.319 ± 0.054	0.121 ± 0.021*
Spleen	0.489 ± 0.029	0.536 ± 0.112	0.558 ± 0.035	0.509 ± 0.093	0.388 ± 0.035*
Liver	13.994 ± 0.384	13.915 ± 1.565	13.69 ± 1.088	13.396 ± 2.201	13.349 ± 1.256
adrenal glands	0.059 ± 0.007	0.056 ± 0.012	0.058 ± 0.022	0.058 ± 0.001	0.068 ± 0.012
Kidney	2.979 ± 0.065	2.656 ± 0.136*	2.966 ± 0.426	2.661 ± 0.129*	3.022 ± 0.127
Day 14	Vehicle	ITRI-148			
		100 mg/kg	300 mg/kg	600 mg/kg	1000 mg/kg
Heart	1.15 ± 0.043	1.139 ± 0.079	1.252 ± 0.229	1.242 ± 0.08	1.306 ± 0.066*
Thymus	0.321 ± 0.055	0.319 ± 0.039	0.314 ± 0.042	0.394 ± 0.041	0.355 ± 0.119
Spleen	0.59 ± 0.025	0.538 ± 0.05	0.629 ± 0.088	0.594 ± 0.066	0.506 ± 0.023
Liver	11.748 ± 1.429	11.06 ± 0.639	12.08 ± 0.721	12.382 ± 0.877	11.072 ± 0.774
adrenal glands	0.072 ± 0.038	0.084 ± 0.059	0.06 ± 0.016	0.07 ± 0.027	0.062 ± 0.009
Kidney	2.894 ± 0.266	2.65 ± 0.021	2.748 ± 0.366	2.759 ± 0.179	2.763 ± 0.229

Weights of major organs were measured at necropsy on Day 7 (end of dosing) and Day 14 (end of recovery). Data are expressed as mean ± SEM in grams (g) (n = 3 per dose group per sampling day). Significant differences from vehicle controls are indicated with an asterisk.
